# Complete chloroplast genome of *Isoetes japonica* (Isoetaceae)

**DOI:** 10.1080/23802359.2021.2005485

**Published:** 2022-11-15

**Authors:** Xiaoyan Lin, Yufeng Gu, Yuehong Yan, Baodong Liu

**Affiliations:** a Key Laboratory of Plant Biology in Colleges of Heilongjiang Province, Life Science and Technology College, Harbin Normal University, Harbin, Heilongjiang, China; b Key Laboratory of National Forestry and Grassland Administration for Orchid Conservation and Utilization, The National Orchid Conservation & Research Center of Shenzhen, Shenzhen, Guangdong, China

**Keywords:** Heterospores, plastome, phylogeny

## Abstract

*Isoetes japonica* A. Braun is a heterosporous quillwort living in low-altitude areas in Japan. In the present study, the complete chloroplast genome of *I. japonica* was assembled and annotated. This chloroplast genome is a circular structure of 145,517 bp in length, comprising a pair of inverted repeat (IR) regions of 13,204 bp each, a large single copy (LSC) region of 91,868 bp, and a small single copy (SSC) region of 27,241 bp. The chloroplast genome contains 135 genes, including 78 protein-coding genes, 36 tRNA genes, and 8 rRNA genes. Phylogenetic analysis showed that *I. japonica* is sister to *I. sinensis* and *I. yunguiensis.* These results provide additional resources for the future studies on *Isoetes* species.

*Isoetes japonica* A. Braun (2n = 66) is a member of heterospores of *Isoetes* L., distributing in low-altitude areas of Japan (Takamiya 1999). *I. japonica* is either a submerged or emerged plant, growing in ponds, streams, and paddy fields (Takamiya et al. 1994). Megaspore ornamentation of this species present as reticulate and microspore surface is smooth (Takamiya 1999).

In the present study, leaf material was obtained from a specimen (voucher: Miyoshi Furuse 57289) collected from Pacang Town, Tochigi City, Tochigi Prefecture, Japan and deposited in China National Herbarium (PE, 01615847). The leaves were sent to Shanghai Majorbio Bio-pharm Technology Co., Ltd. (Shanghai, China) for DNA extraction, and sequencing was performed on an Illumina HiSeq X Ten platform (Illumina, San Diego, CA, USA). The plastid genome was assembled using GetOrganelle v1.7.5 (Jin et al. 2018), and the results were viewed and edited by Bandage v0.8.1 (Wick et al. 2015). Assembled chloroplast genome was annotated by Geneious Prime 2021.0.3 (https://www.geneious.com) (Kearse et al. 2012) with *I. engelmannii* as the reference.

The complete plastid genome sequence of *I. japonica* (GenBank accession: MZ596344) is 145,517 bp in length, containing a large single-copy (LSC) region of 91,868 bp, a small single-copy (SSC) region of 27,241 bp, and a pair of inverted repeats (IRs) of 13,204 bp each. A total of 135 genes were annotated, including 78 protein-coding genes, 36 transfer RNA (tRNA) genes, and 8 ribosomal RNA (rRNA) genes. The overall GC content is 38.0%.

To figure out the phylogenetic position of *I. japonica*, a phylogenetic analysis was carried out with complete chloroplast genomes of 11 *Isoetes* species downloaded from GenBank, and two members of Lycopodiaceae (*Lycopodium clavatum* and *Huperzia serrata*) were set as outgroups. The sequences were aligned using MAFFT v7 in PhyloSuite v1.2.2 (Zhang et al. 2020). A maximum-likelihood (ML) tree was constructed by RAxML v8 with the GTRCAT nucleotide substitution model and 1,000 rapid bootstraps (Stamatakis 2014).

The ML tree indicated that *I. nuttallii* and *I. cangae* are two sole clades. *I. hypsophila* is sister to the clade of *I. sinensis*, *I. yunguiensis*, and *I. japonica* with a support value of 96%, and *I. japonica* was sister to *I. sinensis* and *I. yunguiensis* with a support value of 100% (Figure 1). The above four species form a clade sister to the clade consisting of *I. mattaponica*, *I. graniticola*, *I. engelmannii*, *I. melanospora*, *I. butleri*, and *I. valida*, showing a 100% support value.

**Figure 1.**
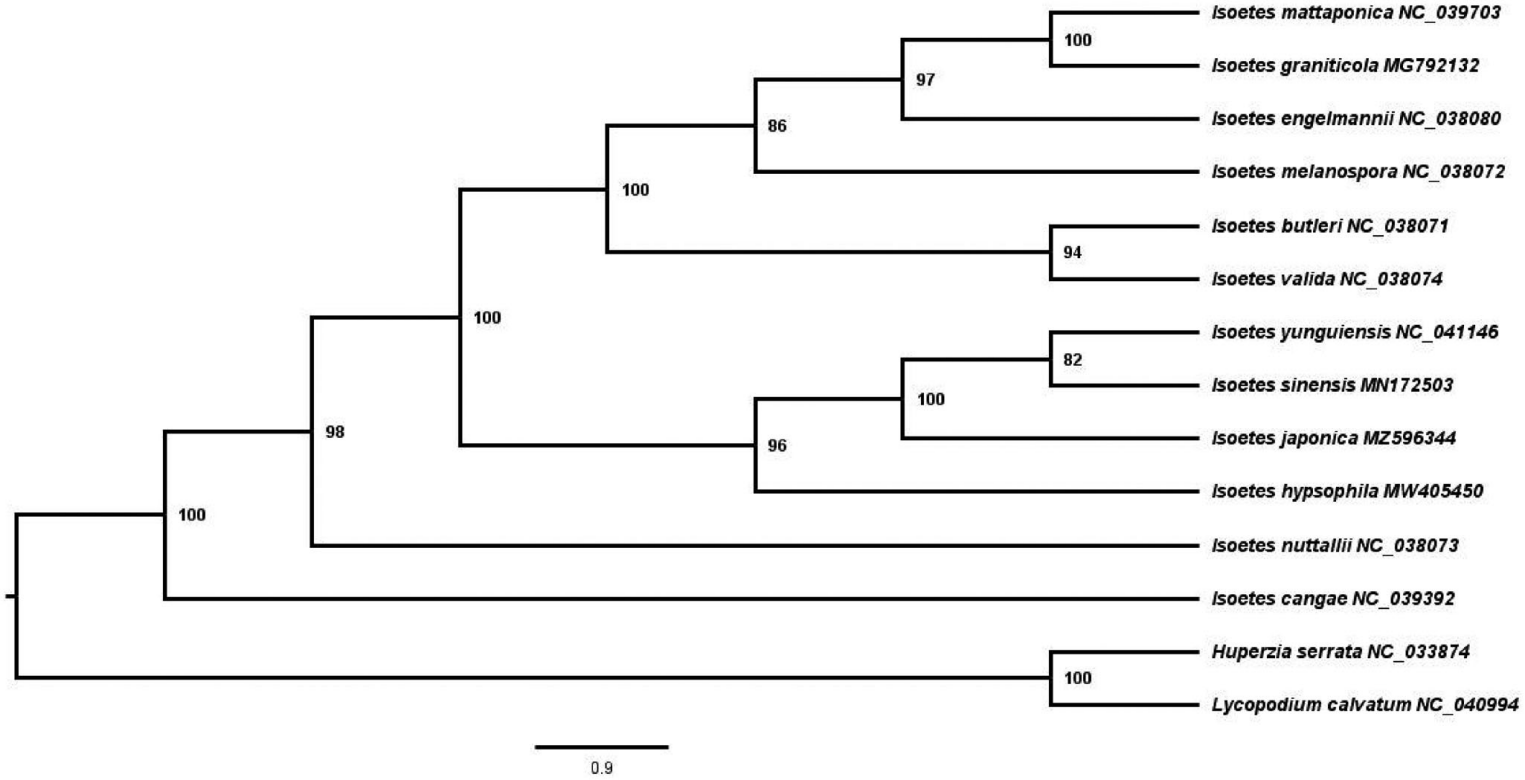
Maximum likelihood phylogenetic tree of 12 *Isoetes* species, with *Lycopodium clavatum* and *Huperzia serrata* as outgroups, constructed based on plastid genome sequences by RAxML. The number next to each node indicates the bootstrap value.

According to the ML tree, *Isoetes japonica* has close relationship to quillworts collected from China, this agree with the view Kim et al. (2010) reported that its speciation may be associated with *I. sinensis* and *I. taiwanensis* but not supported by spore morphology features. Here, our result supports *I. japonica* is the sister clade with *I. sinensis*, we speculate it could possess another speciation pathway. Information presented in this paper and other related data on the net will assist in the future studies of *Isoetes*.

## Data Availability

The genome sequence data that support the findings of this study can be obtained from GenBank of NCBI (https://www.ncbi.nlm.nih.gov/) under the accession no. MZ596344. The associated accession numbers of BioProject, SRA, and Bio-sample are PRJNA731595, SRR15196053, and SAMN20310797, respectively.
